# Surgery for multiple gastric gastrointestinal stromal tumors and large esophageal diverticulum related to germline mutation of the *KIT* gene: a case report

**DOI:** 10.1186/s40792-023-01766-w

**Published:** 2023-10-23

**Authors:** Asami Arita, Tsuyoshi Takahashi, Kiyokazu Nakajima, Yukinori Kurokawa, Seiichi Hirota, Toshirou Nishida, Kotaro Yamashita, Takuro Saito, Koji Tanaka, Tomoki Makino, Makoto Yamasaki, Kunihiko Kawai, Yuichi Motoyama, Eiichi Morii, Hidetoshi Eguchi, Yuichiro Doki

**Affiliations:** 1 Department of Gastroenterological Surgery, Graduate School of Medicine, Osaka University, 2-2 Yamadaoka, Suita City, Osaka 565-0871 Japan; 2 Department of Pathology, School of Medicine, Hyogo Medical University, 1-1 Mukogawa-Cho, Nishinomiya City, Hyogo 663-8501 Japan; 3 Department of Surgery, Japan Community Healthcare Organization, Osaka Hospital, 4-2-78, Fukushima, Fukushima-Ku, Osaka 553-0003 Japan; 4 Department of Pathology, Osaka University Graduate School of Medicine, Osaka University, 2-2 Yamadaoka, Suita City, Osaka 565-0871 Japan

**Keywords:** Familial GIST, Esophageal diverticulum, *KIT* germ-line mutation

## Abstract

**Background:**

Familial gastrointestinal stromal tumors (GISTs) are mesenchymal tumors of the digestive tract caused by germline gain-of-function mutations in the *KIT* gene or platelet-derived growth factor receptor alpha gene (*PDGFRA*). These mutations cause not only multiple GISTs but also diffuse hyperplasia of interstitial cells of Cajal (ICCs), which is related to esophageal motility disorder.

**Case presentation:**

A 53-year-old man was referred to our hospital because of anemia and dysphagia. Fifteen years earlier, he had undergone a laparoscopic partial gastrectomy for multiple gastric GISTs with a germline mutation in exon 17 of the *KIT* gene. An upper gastrointestinal endoscopy revealed that the patient had multiple gastric GISTs and a large esophageal diverticulum directly above the esophagogastric junction. The largest gastric tumor was 7 cm, with a delle that might cause bleeding. Because the patient presented with dysphagia, we performed video-assisted thoracic esophagectomy and laparoscopic-assisted proximal gastrectomy simultaneously. The patient had survived without metastasis for 4 years after surgery and dysphagia had improved.

**Conclusions:**

This is the first report of successful laparoscopic–thoracoscopic surgery for a patient with familial gastric GISTs accompanied with a large esophageal diverticulum.

## Background

Gastrointestinal stromal tumors (GISTs) are mesenchymal tumors of the digestive tract and arise from interstitial cells of Cajal (ICCs) [1], which are the pacemakers of gastrointestinal movement [2]. Familial GISTs are caused by germline gain-of-function mutations in the *KIT* gene or *PDGFRA* gene [3]. These mutations do not cause only multiple GISTs in the GI tract but also diffuse hyperplasia of ICCs, skin pigmentation, mastocytomas, and dysphagia owing to ICC function disorder [3]; however, a case of GISTs accompanied with a large esophagus diverticulum was extremely rare.

Herein, we report a case of a patient familial GISTs accompanied with a large epiphrenic esophageal diverticulum who was successfully treated using video-assisted laparoscopic–thoracoscopic esophagectomy (VATS-E) with gastric conduit reconstruction by hand-assisted laparoscopic surgery (HALS).

## Case presentation

**Fig. 1:**
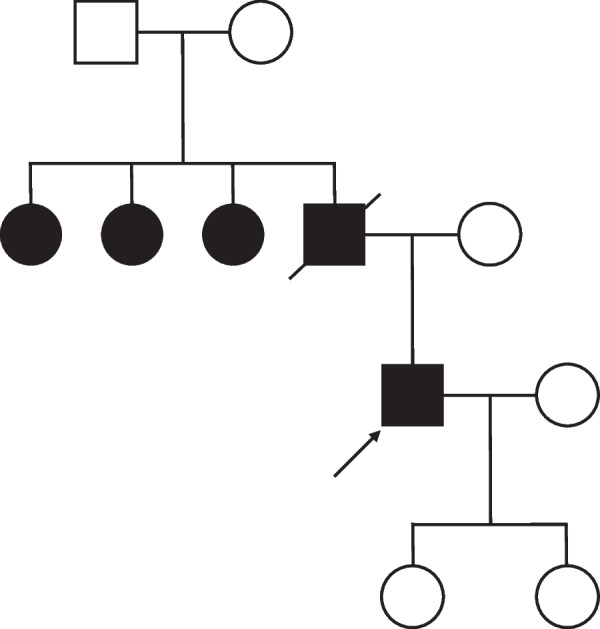
KIT-associated family history. A male is represented by a square and a female by a circle. The arrow indicates this case, the male proband. Black shapes indicate *KIT* mutations. The patient’s father and aunts had GISTs with *KIT* gene mutations

**Fig. 2:**
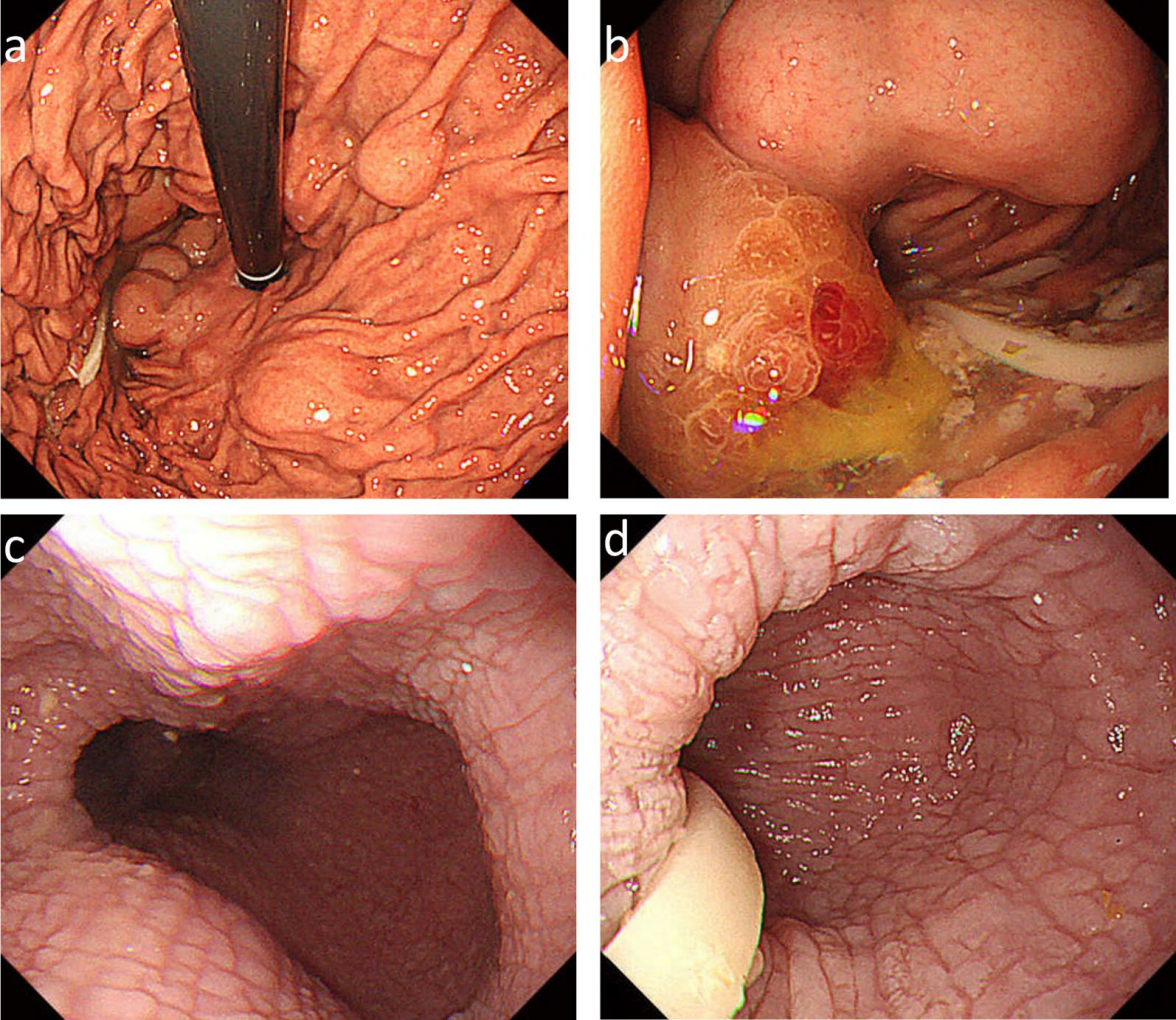
Esophagogastroduodenoscopy revealed multiple GISTs directly below the cardia and the dome of the stomach (**a**). The largest GIST had a delle, which seemed to cause bleeding (**b**). There was a large esophageal diverticulum immediately above the esophagogastric junction (**c**). A quantity of undigested food remained in the esophageal diverticulum (**d**)

**Fig. 3:**
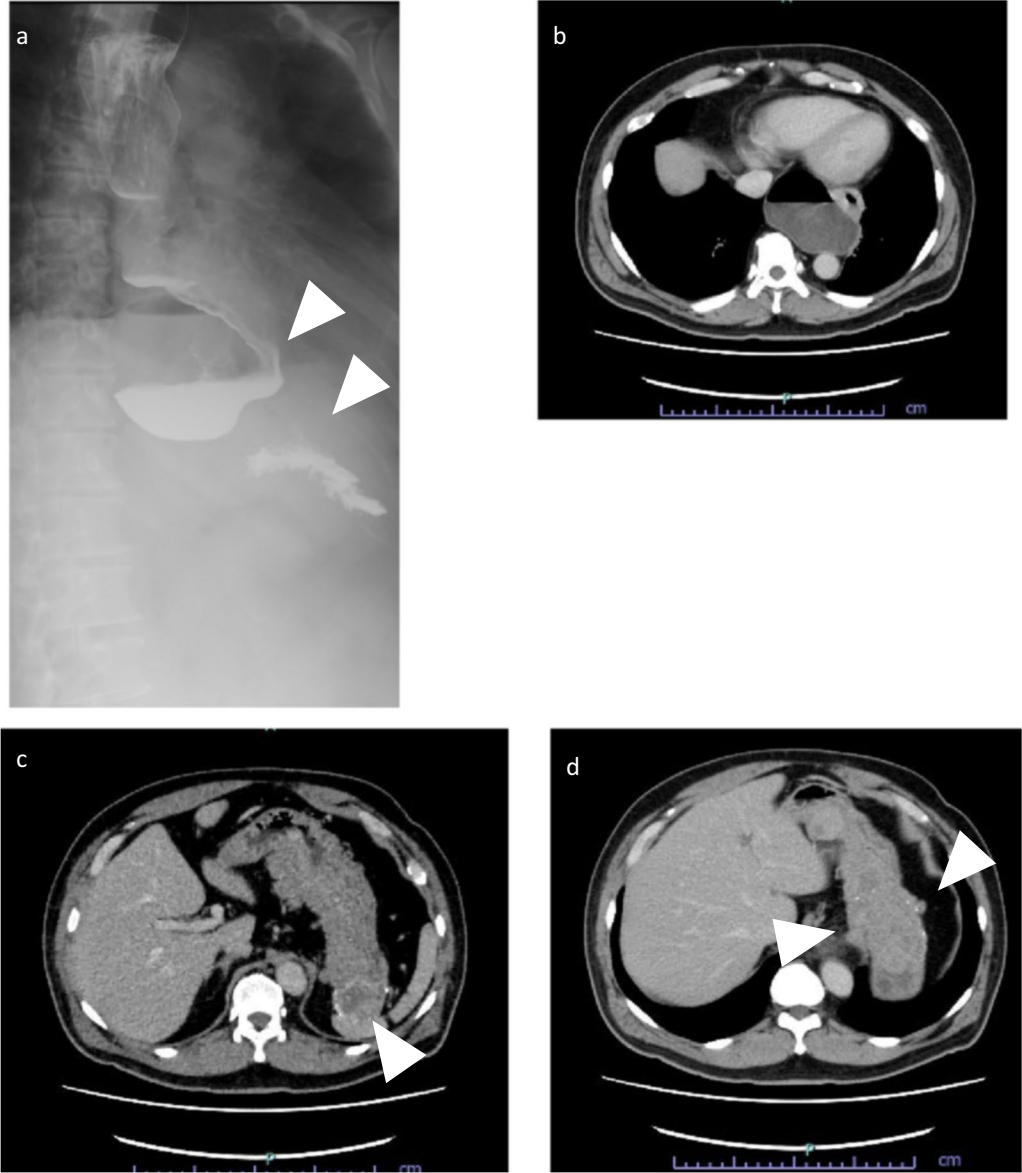
Gastrointestinal series contrast medium did not flow into the stomach smoothly (white arrow heads) because of the large esophageal diverticulum (**a**). CT images of the esophageal diverticulum (**b**), gastric large GIST (white arrow head) (**c**) and multiple GISTs (white arrow heads) (**d**)

The patient was a 53-year-old man who had undergone laparoscopic partial gastrectomy for multiple GISTs in the fornix with a germline mutation in exon 17 of the *KIT* gene in our hospital 15 years earlier. The GISTs were accidently found by esophagogastroduodenoscopy at screening, and the operation was performed because of the increasing size of the tumors for some months. There was not esophageal diverticulum at that time. The patient’s father and aunt also had GISTs with the same mutation (Fig. 1). In addition, he showed esophageal motor dysfunction by the esophageal manometry. After the operation, annual follow-ups were conducted by another hospital. Fifteen years later, the patient was referred to our hospital for gastric tumor bleeding, and the patient had a significantly low hemoglobin level at 5.0 g/dl. After esophagogastroduodenoscopy, the patient was diagnosed with multiple GISTs, with an ulcer directly below the cardia and fornix of the stomach (Fig. 2a, b) and a large esophageal diverticulum directly above the esophagogastric junction (Fig. 2c, d). The esophageal diverticulum contained undigested food, making it difficult to reach the stomach. Upper gastrointestinal series showed obstruction of the barium outflow because of the large esophageal diverticulum (Fig. 3a). Chest and abdomen computed tomography confirmed the presence of the diverticulum (Fig. 3b) and multiple GISTs (Fig. 3c, d). No other intestinal tumors nor disseminations were detected in the abdominal cavity. Because the patient experienced dysphagia due to the obstruction by the large esophageal diverticulum, we decided to perform esophagectomy and proximal gastrectomy by a thoracoscopic–laparoscopic approach simultaneously.

**Fig. 4:**
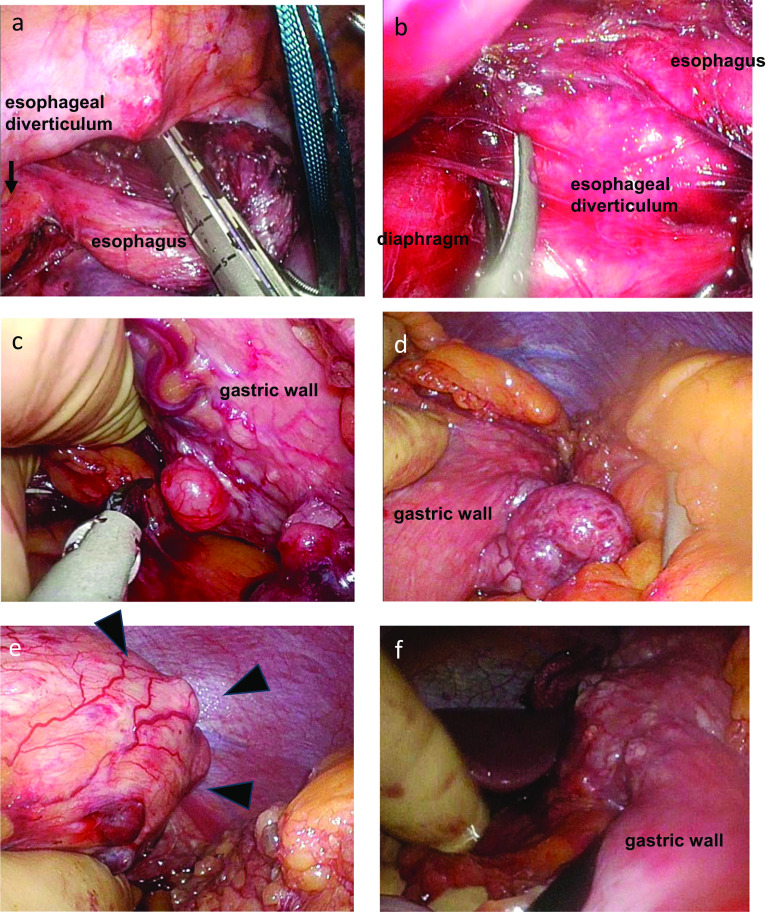
Operative findings: we dissected the thoracic esophagus immediately above the diverticulum to avoid damaging the diverticulum (black arrow) (**a**). After cutting the esophagus, we dissected around the esophagus hiatus (**b**). By the HALS, we performed proximal gastrectomy. We confirmed multiple extramural growth GISTs in the lesser curvature of gastric wall (**c**), and in the greater curvature of gastric wall (**d**). We palpated the largest GIST which grew luminally in the fornix (black arrow heads) (**e**). The presence of more than ten small GISTs (<1 cm) was identified in the remaining stomach (**f**)

We performed thoracic procedures from right side approach. We started cutting the parietal pleurae and dissected the thoracic esophagus just above the diverticulum, putting a vessel tape around the esophagus. We cut the esophagus using the Signia™ Stapling System (Covidien, Dublin, Ireland) in the position of the azygous vein (Fig. 4a) and peeled off to the posterior to prevent damage to the diverticulum (Fig. 4b). In the abdominal procedures, we chose HALS to avoid the risk of tumor rupture. We opened the bursa omentalis and cut the omentum to the either side, cutting the left gastroepiploic artery and vein and left gastric artery. We peeled around the esophagus hiatus, pulling the thoracic esophagus into the abdominal cavity. We resected the abdominal esophagus and proximal stomach, including numerous GISTs (Fig. 4c–e). The presence of more than ten small GISTs (< 1 cm) was identified in the remaining stomach (Fig. 4f). Residual GISTs necessitated subcutaneous reconstruction with a gastric conduit, because we might have to perform gastrectomy in the future due to regrowth of the remaining GISTs. The surgical time was 479 min, and volume of intraoperative blood loss was 440 ml. He had no postoperative complications and was discharged from hospital on the 24th postoperative day. Postoperatively, the symptoms of dysphagia due to a large esophageal diverticulum disappeared, and the patient had survived without regrowth, local recurrence, or distant metastasis of remaining GISTs for 4 years after surgery.

**Fig. 5:**
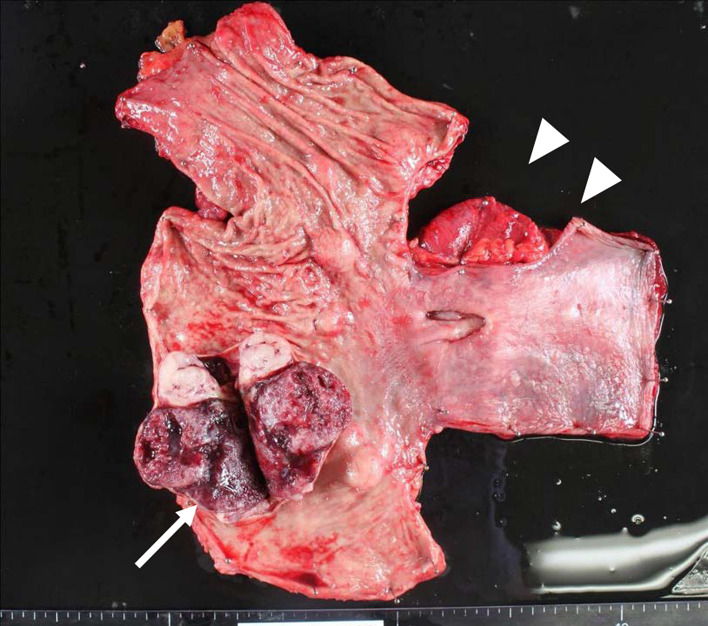
Largest of the gastrointestinal tumors was 70 × 60 × 50 mm (arrow), and we confirmed the presence of a large esophageal diverticulum (arrow heads)

**Fig. 6:**
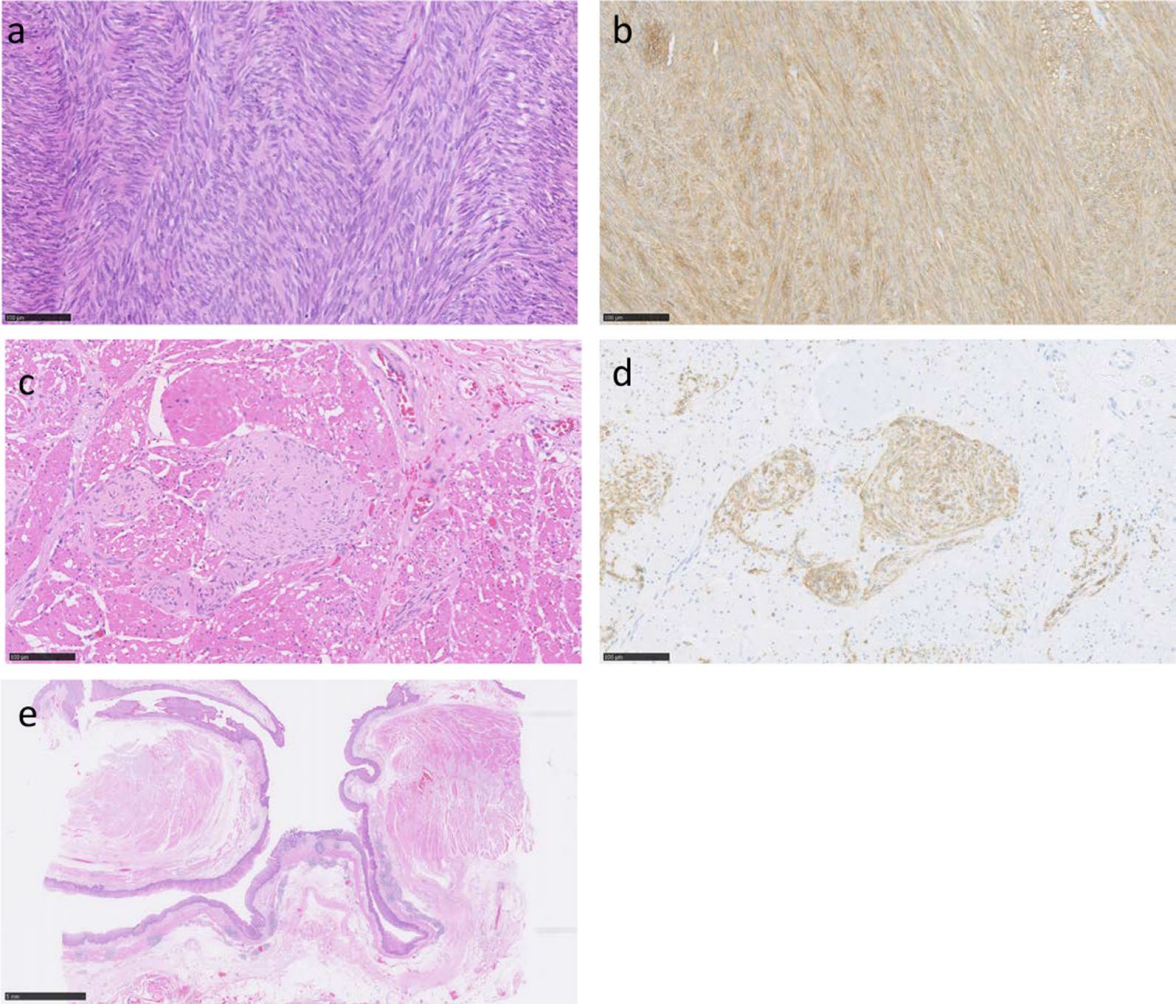
Pathological findings. Bar represents 100 μm. Hematoxylin and eosin (**a**) and KIT (**b**) sections of gastric GISTs. Hematoxylin and eosin (**c**) and KIT (**d**) sections of the esophageal muscle. We confirmed the hyperplasia of ICC, a typical image of familiar GISTs. Hematoxylin and eosin section of esophageal diverticulum (**e**)

The resected specimen revealed that the largest GIST was 70 × 60 × 50 mm, and 10 other small GISTs were existed. We confirmed a 10 cm diverticulum arising from the lower esophagus (Fig. 5). From histological examination, the tumors were mainly based on muscular propria and were KIT positive in immunohistochemistry staining (Fig. 6a, b). There were five more nuclear divisions, < 10 per 50 high power fields, and MIB-1 index was 2–3%. By the genetic test, they have a same mutation in exon 17 of the *KIT* gene. Furthermore, there were numerous images of ICC hyperplasia observed on the muscle layer of the resected esophageal tissue (Fig. 6c, d). The large esophageal diverticulum was proved to be a pseudodiverticulum (Fig. 6e).

## Discussion

GISTs are mesenchymal tumors of the digestive tract and are thought to arise from ICCs [4]. Gain-of-function mutations of the *KIT* or *PDGFRA* gene in the ICCs cause ligand-independent activation of the corresponding receptor for tyrosine kinase and subsequently activate common downstream signaling pathways. *KIT* mutations in sporadic GISTs, which account for 80–90% of all GISTs, have been reported in exon 9, 11, 13, and 17 of the gene. The mutation in exon 11 accounts for more than 50%, the mutation in exon 9 is found in 10–20%, and mutations in exons 13 and 17 are rarely found [5, 6]. ICCs are innervated cells associated with Auerbach’s plexus that regulate gastrointestinal (GI) motility and coordinate peristalsis throughout the GI tract. Because ICCs are present throughout in the gastrointestinal wall, GISTs can occur anywhere, especially in the stomach (50%) and small bowel (25%) [4, 7].

After the first report by Nishida et al. [8], over 40 families who have multiple family members developing GIST have been identified. Familial GIST is an inherited neoplastic disease with multiple GISTs throughout the GI tract caused by germline mutations in *KIT* gene or *PDGFRA* gene [9, 10]. In familial GISTs, 55% of families had mutations in exon 11 of the *KIT* gene and 20% of families had mutations in exon 13 of the *KIT* gene [8, 9]. The median age of patients with familial GISTs is lower than that of patients with sporadic GISTs. Hyperplasia of ICC is observed histologically, resulting in acquisition of other gene mutations, development of monoclonal GIST, and occurrence of malignant transformation of tumors. Multiple GISTs have been observed in the small intestine and stomach, but rarely in the colon [8, 9]. Clinically, it is important not to misdiagnose multiple GISTs as peritoneal metastasis.

These mutations cause not only multiple GISTs but also diffuse ICC hyperplasia in GI tract, skin pigmentation, vitiligo, mastocytomas, and dysphagia due to ICC function disorder in the esophagogastric junction. In the report by Hirota et al., the hyperplasia of ICCs in the myenteric plexus region of the esophagus was observed pathologically and endoscopic ultrasound of the esophagocardiac junction showed a thickened hyperechoic layer between the circular and longitudinal muscle layers without mechanical obstruction of the esophagus [8–10].

Abnormalities of ICCs have been implicated in various motility disorders of the GI tract [11, 12]. The disordered motility associated with ICC hyperplasia might lead to decreased peristalsis, cause achalasia-like disorder [11], and predispose patients to diverticula formation. This patient and his father had a germline mutation in exon 17 of the *KIT* gene and had also multiple GISTs and esophageal motility disorder. Furthermore, Shintaku et al. reported the relationship between GISTs and diverticular structure [13]. The study stated that GISTs might locally alter gut motility and induce disturbed peristalsis. In the present case, numerous familial GISTs in stomach also can alter gut motility and result in food residue accumulation in esophagus, with extension of the lumen of the esophagus forming the large esophageal diverticulum.

Considering about the operative procedure in this case, there are some other procedures, such as resection of the diverticulum preserving the esophagus. However, the location of the diverticulum was near from the esophageal gastric junction and it is difficult to resect only the diverticulum. In addition, because this patient has the esophageal motility disorder genetically, it may cause esophageal reflux symptom in the future and we decided to this operative method. Furthermore, we examined the patient’s abdominal cavity laparoscopically and confirmed multiple small tumors throughout the stomach. However, because the patient was predicted to have numerous GISTs throughout the GI tract and he wished to avoid total gastrectomy, we decided to perform proximal gastrectomy and to perform reconstruction via the subcutaneous route for future possibility of re-gastrectomy. Laparoscopic–thoracoscopic esophageal resection for the treatment of large epiphrenic esophageal diverticulum has been previously reported [14]; however, this is the first report of laparoscopic–thoracoscopic surgery for not only a large esophageal diverticulum but also for familial GISTs.

## Conclusion

We encountered a rare case of a familial GIST with a large esophageal diverticulum.

Laparoscopic–thoracoscopic approach might be an effective approach as a definitive minimally invasive therapy.

## Data Availability

Data sharing is not applicable to this article, as no data sets were generated or analyzed during the current study.

## Abbreviations

GIST: Gastrointestinal stromal tumors
PDGFRA: Platelet-derived growth factor receptor alpha gene
ICC: Interstitial cells of Cajal
VATS-E: Video-assisted laparoscopic–thoracoscopic esophagectomy
HALS: Hand-assisted laparoscopic surgery
